# Effects of diesel exposure on lung function and inflammation biomarkers from airway and peripheral blood of healthy volunteers in a chamber study

**DOI:** 10.1186/1743-8977-10-60

**Published:** 2013-12-09

**Authors:** Yiyi Xu, Lars Barregard, Jörn Nielsen, Anders Gudmundsson, Aneta Wierzbicka, Anna Axmon, Bo AG Jönsson, Monica Kåredal, Maria Albin

**Affiliations:** 1 Division of Occupational and Environmental Medicine, Lund University, SE 221-85 Lund, Sweden; 2 Department of Occupational and Environmental Medicine, Sahlgrenska University Hospital and Academy, University of Gothenburg, Box 414, SE-405 30 Gothenburg, Sweden; 3 Department of Occupational and Environmental Medicine, Division of Laboratory Medicine, Region Skåne, Sweden; 4 Ergonomics and Aerosol Technology, Lund University, Box 118, SE-221 00 Lund, Sweden

**Keywords:** Diesel exhaust, Chamber experiment, Lung function, Biomarkers, Respiratory inflammation

## Abstract

**Background:**

Exposure to diesel exhaust causes inflammatory responses. Previous controlled exposure studies at a concentration of 300 μg/m^3^ of diesel exhaust particles mainly lasted for 1 h. We prolonged the exposure period and investigated how quickly diesel exhaust can induce respiratory and systemic effects.

**Methods:**

Eighteen healthy volunteers were exposed twice to diluted diesel exhaust (PM_1_ ~300 μg/m^3^) and twice to filtered air (PM_1_ ~2 μg/m^3^) for 3 h, seated, in a chamber with a double-blind set-up. Immediately before and after exposure, we performed a medical examination, spirometry, rhinometry, nasal lavage and blood sampling. Nasal lavage and blood samples were collected again 20 h post-exposure. Symptom scores and peak expiratory flow (PEF) were assessed before exposure, and at 15, 75, and 135 min of exposure.

**Results:**

Self-rated throat irritation was higher during diesel exhaust than filtered air exposure. Clinical signs of irritation in the upper airways were also significantly more common after diesel exhaust exposure (odds ratio=3.2, p<0.01). PEF increased during filtered air, but decreased during diesel exhaust exposure, with a statistically significant difference at 75 min (+4 L/min vs. -10 L/min, p=0.005). Monocyte and total leukocyte counts in peripheral blood were higher after exposure to diesel exhaust than filtered air 20 h post-exposure, and a trend (p=0.07) towards increased serum IL-6 concentrations was also observed 20 h post-exposure.

**Conclusions:**

Diesel exhaust induced acute adverse effects such as symptoms and signs of irritation, decreased PEF, inflammatory markers in healthy volunteers. The effects were first seen at 75 min of exposure.

## Background

The adverse health effects of particulate air pollution are well documented [1-5], and urban outdoor air pollution was estimated to cause 1.34 million premature deaths worldwide in 2008, an increase of 16% from 2004 [6]. Diesel exhaust (DE) emissions constitute, according to estimates from the USA, 4-16% of the total particulate matter (PM) in non-urban and urban areas [7,8]. People working around diesel equipment or living close to major roadways are more likely to have irritant respiratory symptoms and reduced baseline pulmonary function [9,10], and to develop respiratory disease including allergy [11], chronic obstructive pulmonary disease [12,13], and lung cancer [14-16].

Epidemiological studies have shown associations between DE and respiratory diseases, but the underlying mechanisms are still not fully understood and the exposure needs to be better characterized to improve the basis for risk assessment in occupational and environmental settings. Part of this knowledge gap was filled by a series of controlled human diesel exhaust exposure studies carried out in Umeå, Sweden. They monitored exposure, and investigated the mechanisms behind the observed effects. Healthy and asthmatic volunteers were exposed in an exposure chamber to either DE concentrations of 300 μg/m^3^ (measured as PM_10_) for 1 h [17-19] or 100 μg/m^3^ for 2 h [20,21]. The results demonstrated that DE exposure caused inflammatory responses at 6 h and 18 h. This was indicated by increased cell counts (neutrophils, B lymphocytes and eosinophils) and concentrations of fibronectin and methyl histamine in airway lining fluid, as well as increase neutrophils and mast cells, up-regulation of endothelial adhesion molecules and interleukins in bronchial biopsies [17-19,22]. In one experiment, this airway inflammation was also followed by an increase of neutrophils and platelets in peripheral blood [19].

Additionally, a DE-induced bronchoconstrictive response (increased airway resistance and specific airway resistance) was observed as measured by computerized whole body plethysmography [20,23,24]. However, no significant change was detected with conventional pulmonary function tests (dynamic spirometry and peak expiratory flow) [19,25].

The Umeå studies have provided extensive knowledge on the respiratory effects of moderate DE exposure. However, the absence of lung function changes in conventional tests in most of the Umeå studies may be due to the relatively short exposure time and follow-up time. In the present investigation, which is a part of the DINO study “Health Effects of Combined Exposure to Diesel and Noise”, we prolonged the exposure time to 3 h and monitored symptoms and peak expiratory flow (PEF) over the whole exposure period. The aim was to find potential relationships between exposure and effects, and to monitor how quickly such changes would develop.

## Materials and methods

### Subjects

Eighteen healthy, non-smoking volunteers (9 males and 9 females) with a mean age of 51 (range 40–66) years were recruited. Inclusion criteria were no symptoms or diagnosis of asthma, normal chest auscultation, lung function and a negative skin prick test to a standard panel of aeroallergens for atopy. The study was approved by the Regional Ethical Review Board, and all subjects provided written consent.

### Exposure protocol

Each subject underwent four different exposure scenarios at rest (seated quietly): (1) Reference exposure: low DE concentration (measured as mass concentration of particles with an aerodynamic diameter less than 1 μm, PM_1_ ~2 μg/m^3^) and low traffic noise (46 dB(A)); (2) Diesel exposure: high DE concentration (PM_1_ ~300 μg/m^3^) and low traffic noise; (3) Noise exposure: low DE concentration and high traffic noise (75 dB(A)); and (4) Diesel and noise exposure: high DE concentration and high traffic noise. Exposure lasted for 3 h and took place in a specially built exposure chamber (22 m^2^, suitable for 3 persons simultaneously) on different occasions in a randomized sequence, at least one week apart. The study design was double-blind, however, the difference between high and low traffic noise was easily noticed by study subjects and the physician.

In this paper, the reference exposure and the noise exposure were merged and renamed “filtered air” (FA) exposure, while the diesel exposure and the diesel and noise exposure were merged and renamed “diesel exhaust” (DE) exposure, since no interactions were found between DE and noise in any marker of respiratory effects (see Statistical analysis section).

The DE was generated from a passenger car (Volkswagen Passat TDI, -98, 1900 cm^3^, 81 kW) running in idle mode. Swedish Environmental Class 1 diesel fuel with a sulfur content less than 10 ppm was used. Dilution was controlled to supply the desired concentration of particles to the chamber for the different exposure scenarios. Details of diesel aerosol generation, as well as DE characteristics in both particle and gas phase are reported elsewhere [26]. Traffic noise recorded at a street crossing was played from two loudspeakers reproducing each channel in a stereo recording. The noise had a continuous, natural change of noise level. The temperature and the relative humidity in the chamber were maintained at 23°C and between 30% and 40%, respectively. Characteristics of DE and FA exposures are presented in Table 1.

**Table 1:** Exposure details during DE and FA exposures

	**DE exposure**	**FA exposure**
PM_1_ mass concentration (μg/m^3^)	276±27	2±2
Number concentration (particles/cm^3^)	388000±48000	14±16
NO (ppm)	9.8±1.0	0.003±0.001
NO_2_ (ppm)	1.3±0.4	0.002±0.001
CO_2_ (ppm)	1945±125	947±67
Formaldehyde (μg/m^3^)	400±97	7.5
Temperature (°C)	22.7±0.7	22.7±0.8

Values are mean ± standard deviation (SD).

*DE*, diesel exhaust; *FA*, filtered air.

### Self-reported symptoms and medical examinations

The questions about symptoms of upper airways, including runny nose, nasal congestion, throat irritation and chest tightness, were rated on a visual analogue scale (VAS) (range 0 to 100 millimeter) before exposure, and at 15, 75 and 135 min into exposure during each exposure session. The VAS is most appropriate at repeated measurement within subjects as it has the sensitivity required to measure minute changes [27]. Signs of nose and throat irritation (redness/secretion/swelling) were recorded (normal/slight/moderate/pronounced), and auscultation of lungs was performed in a medical examination before and after exposure by the same physician. For scheduling of examinations and samplings see Table 2.

**Table 2:** **Scheduling and time point of different examinations**, **blood and nasal lavage samplings**

**Item**	**Before exposure (outside chamber)**	**During exposure (in chamber)**			**After exposure (outside chamber)**	**20 h post-exposure (outside chamber)**
		**15 min**	**75 min**	**135 min**		
Self-rating symptoms	× (7:40)	× (9:45)	× (10:45)	× (11:45)		
Medical examination	× (7:00)				× (13:10)	
Peak expiratory flow	× (7:40)	× (9:45)	× (10:45)	× (11:45)		
Rhinometry	× (8:30~9:00)				× (13:20~13:50)	
Spirometry	× (8:30~9:00)				× (13:20~13:50)	
Blood samples	× (8:30~9:00)				× (13:20~13:50)	× (7:00~8:30)
Nasal lavage	× (8:30~9:00)				× (13:20~13:50)	× (7:00~8:30)

### Lung function and nasal patency

PEF was measured in two ways: (1) PEF data output from a Mini-Wright Peak Flow Meter (MWPFM) with a measuring range of 60–800 L/min, which was measured four times (three recordings each time) in each exposure session: before exposure and at 15, 75 and 135 min into exposure; (2) PEF data output from a computerized spirometer (SPIRARE 3, DIAGNOSTICA, Oslo, Norway), which was measured only before and after exposure (three recordings each time).

Rhinometry and spirometry (a minimum of three recordings for each outcome) were performed before and after exposure: forced vital capacity (FVC) and forced expiratory volume in one second (FEV_1_) were measured according to the European Respiratory Society guidelines [28], using the computerized spirometer. The minimal cross-sectional area between 0–22 mm (MCA_1_) and between 22–54 mm (MCA_2_) into the nasal opening, and the volume of the nasal cavity between these distances (Volume_1_ and Volume_2_) were determined using acoustic rhinometry (RhinoScan v. 2.5, Interacoustics A/S, Assens, Denmark) [29]. The “best” results at each time point from PEF, spirometry and acoustic rhinometry measurements were included in the statistical analysis.

### Nasal lavage

Nasal lavage was performed in this study since it has been proposed as a noninvasive method to detect upper airway inflammation of air pollutants [30,31]. We conducted nasal lavage before exposure, after exposure, and at 20 h post-exposure (range 18–22 h), with minor modifications as described by Benson et al. [32]. Briefly, 18 ml 37°C 0.9% NaCl solution was instilled with a syringe into one nostril until the liquid appeared in the opposite one. The liquid was sucked back and instilled again. The procedure was repeated three times in each nostril. The lavage liquid was immediately chilled and the cells were separated by centrifugation (Centrifuge 5702R, Eppendorf AG, Hamburg, Germany). The supernatant was separated from the pellet and stored at -80°C until analysis of inflammatory markers.

### Inflammatory markers in blood and nasal lavage

Peripheral blood samples (before exposure, after exposure, 20 h post-exposure) were analyzed for total and differential leukocyte cell counts (Sysmex XE-1800i). Plasma fibrinogen (Sysmex CA-7000) and C reactive protein (CRP, Cobas c501) concentrations were determined.

The serum concentrations of interleukin-6 (IL-6) and IL-8, and the nasal lavage concentrations of IL-6, IL-8, intercellular adhesion molecule-1 (ICAM-1) and tumor necrosis factor-α (TNF-α) were analyzed by multiplexed magnetic bead-based immunoassays on a Luminex platform (Bio-Plex 200, Bio-Rad Life Science, Hercules, CA).

Clara cell protein (CC16) and surfactant protein D (SP-D) in serum were analyzed using commercial ELISA kits from Biovendor (BioVendor Laboratory Medicine, Inc., Brno, Czech Republic), according to the protocols supplied by the manufacturer. The imprecision, expressed as coefficient of variation, was 13% for CC16 and 12% for SP-D, as calculated from duplicate analyses.

### Statistical analysis

Absolute changes from baseline were calculated for each individual and each exposure scenario to reduce the influence of inter-individual variation for the data from self-rated symptoms, lung function, biomarkers in blood and nasal lavage. Then the four exposure scenarios were merged into two, as explained above, since the effect of noise did not modify the effect of DE in any of the markers (p>0.06 for all); the variation of the mean PM_1_ mass concentrations between all DE exposures (i.e. the diesel exposures and the diesel and noise exposures) from the mean value of 276 μg/m^3^ (Table 1) was on average smaller than 6.5%. The changes in the selected outcome measures at DE exposure vs. changes at FA exposure were analyzed with repeated-measures analysis of variance using a linear model type of the generalized estimating equation in SPSS 18.0 (SPSS Inc., Chicago, IL, USA). Subject identification, the four initial exposure scenarios, and the time point of measurements were used to indicate the repeated measurements. Exposure sequence was not included in the final models since inclusion of the exposure sequence did not change the results.

The data from the medical examination was transformed to binary variables. If signs (redness/secretion/swelling of nose, throat, and sound of lung and heart auscultation) after exposure became worse than before exposure (from normal to slight, and so on), we recorded it as “getting worse”, otherwise “no change”. Any “getting worse” sign of nose and throat, or sound of lungs represented a corresponding positive finding of upper-airway irritation or lung signs. A binary logistic model type of generalized estimating equation was used to estimate the odds ratios.

Statistical significance refers to p<0.05 (two-tailed). However, multiple comparisons were performed for self-rated symptoms (three times), PEF from MWPFM (three times), and inflammatory markers in blood and nasal lavage (two times). For those comparisons, statistical significance refers to p<0.017 and p<0.025, respectively, according to the Bonferroni correction.

## Results

### Symptoms and Signs

Scores of self-rated symptoms are presented in Table 3. Subjects reported a mild but progressive irritation in the throat during DE exposure, the increment on the VAS scale was 3.75 in DE exposure versus -1.53 in FA exposure (p=0.016) at 135 min. No significant differences between exposures were detected in self-rated nose or chest symptoms.

**Table 3:** **Effects of FA exposure and DE exposure on self**-**rated symptoms**

	**Before exposure**	**15 min exposure**	**75 min exposure**	**135 min exposure**	**Δ 15 min**^**a**^	**Δ 75 min**^**a**^	**Δ 135 min**^**a**^
***FA exposure***							
Runny nose	1.39±7.83	0.08±0.5	0.14±0.83	0.00±0.00	-1.31±7.86	-1.25±7.88	-1.39±7.83
Nasal congestion	1.69±7.99	0.53±1.18	0.61±1.87	0.36±1.36	-1.17±8.12	-1.08±8.21	-1.33±8.11
Throat irritation	1.66±7.46	1.00±5.01	0.11±0.46	0.08±0.50	-0.61±8.64	-1.50±7.03	-1.53±7.36
Chest tightness	--^*^	--^*^	--^*^	0.14±0.83	--^*^	--^*^	0.14±0.83
***DE exposure***							
Runny nose	2.86±10.91	1.97±10.09	1.44±7.66	1.69±8.34	-0.89±3.44	-1.42±4.86	-1.17±4.31
Nasal congestion	3.03±10.55	1.72±7.52	2.69±8.08	3.53±9.32	-1.31±4.33	-0.33±8.10	0.50±6.98
Throat irritation	2.31±6.96	1.06±2.27	4.00±8.64	6.06±12.11	-1.25±6.21	1.69±10.32	3.75±12.11^#^
Chest tightness	0.28±1.67	0.17±0.74	0.17±0.74	0.56±2.29	-0.11±1.51	-0.11±1.85	0.28±2.88

Values are mean ± standard deviation (SD) using a visual analog scale.

*DE*, diesel exhaust; *FA*, filtered air.

^a^Δ 15 min = 15 min of exposure – before exposure; Δ 75 min = 75 min of exposure – before exposure; Δ 135 min = 135 min of exposure – before exposure.

^*^No symptoms were reported. ^#^P <0.017 for DE exposure vs. FA exposure.

Irritation in upper airways (nose and throat) was found in the physical examination. Exposure to DE increased the odds ratio (OR) of signs in the upper-airways (OR=3.2, 95% CI 1.9-11.0). No change was found in lung sounds (data not shown).

### Lung function

Similar PEF values at baseline were obtained with the MWPFM and the spirometer for both FA and DE exposure (Table 4). However, after 15 min of exposure, PEF decreased during DE exposure but increased during FA exposure, and this pattern lasted throughout the whole exposure period. The difference between PEF changes in DE and FA was 14 L/min at 75 min of exposure (MWPFM, p=0.005), 12 L/min at 135 min of exposure (MWPFM, p=0.020) and 10 L/min after exposure (spirometer, p=0.078) (Table 4).

**Table 4:** Effects of FA exposure and DE exposure on PEF as measured using a MWPFM and a SPIRARE spirometer

	**Before exposure**	**15 min exposure**	**75 min exposure**	**135 min exposure**	**After exposure**	**Δ 15 min**^**a**^	**Δ 75 min**^**a**^	**Δ 135 min**^**a**^	**Δ after exosure**^**a**^
***FA exposure***									
PEF (L/min) from MWPFM	530±73	533±73	534±73	538±75	--^*^	3±25	4±26	8±31	--^*^
PEF (L/min) from spirometer	524±88	--^*^	--^*^	--^*^	531±90	--^*^	--^*^	--^*^	8±27
***DE exposure***									
PEF (L/min) from MWPFM	537±74	531±75	528±78	534±81	--^*^	-6±22	-10±22 ^#^	-4±18	--^*^
PEF (L/min) from spirometer	530±93	--^*^	--^*^	--^*^	527±89	--^*^	--^*^	--^*^	-3±24

Values are mean ± standard deviation (SD).

*DE*, diesel exhaust; *FA*, filtered air; *PEF*, peak expiratory flow; *MWPFM*, Mini-Wright Peak Flow Meter.

^a^Δ 15 min = 15 min of exposure – before exposure; Δ 75 min = 75 min of exposure – before exposure; Δ 135 min = 135 min of exposure – before exposure; Δ after exposure = after exposure – before exposure.

^*^No PEF was recorded. ^#^P <0.017 for DE exposure vs. FA exposure.

The changes in spirometry (FEV_1_, FVC) and rhinometry results were small and similar after both DE and FA exposure (Table 5).

**Table 5:** **Effects of FA exposure and DE exposure on spirometry** (**FEV**_**1**_, **FVC**) **and rhinometry** (**MCA**_**1**_, **MCA**_**2**_, **Volume**_**1**_, **Volume**_**2**_)

		**Before exposure**	**After exposure**	**Δ after exposure**^**a**^
**Spirometry**				
FEV_1_	FA exposure	3.23±0.57	3.26±0.58	0.02±0.13
	DE exposure	3.23±0.60	3.26±0.59	0.03±0.08
FVC	FA exposure	4.12±0.71	4.14±0.70	0.02±0.16
	DE exposure	4.13±0.72	4.17±0.72	0.04±0.10
**Rhinometry**				
MCA_1_	FA exposure	1.12±0.28	1.13±0.23	0.01±0.18
	DE exposure	1.08±0.24	1.13±0.24	0.05±0.16
MCA_2_	FA exposure	0.95±0.23	1.06±0.25	0.11±0.19
	DE exposure	0.94±0.20	1.03±0.31	0.10±0.23
Volume_1_	FA exposure	4.06±0.75	4.07±0.67	0.01±0.26
	DE exposure	4.05±0.72	4.10±0.69	0.05±0.21
Volume_2_	FA exposure	6.90±1.56	8.16±1.59	1.26±1.74
	DE exposure	6.51±1.19	7.82±1.93	1.32±1.58

Values are mean ± standard deviation (SD).

*DE*, diesel exhaust; *FA*, filtered air; *FEV*_*1*_, forced expiratory volume in one second; *FVC*, forced vital capacity; *MCA*_*1*_, minimal cross-sectional area between 0–22 mm; *MCA*_*2*_, minimal cross-sectional area between 22–45 mm.

^a^Δ after exposure = after exposure – before exposure.

### Inflammatory markers

There was a significantly greater increment of total leukocytes in blood at 20 h post-exposure following DE exposure compared to FA exposure (Table 6, p=0.007), including a significantly higher increment of monocyte counts (p=0.017). A trend towards an increase in monocyte counts between DE exposure and FA exposure was suggested also immediately after exposure (p=0.04). No such patterns were discerned for neutrophils, eosinophils or lymphocytes.

**Table 6:** Effects of FA exposure and DE exposure on cell count in peripheral blood

**Cell type**	**Cell count (×10**^**9**^**/L)**					**P-values for DE exposure vs. FA exposure**	
	**Before exposure**	**After exposure**	**20 h post-exposure**	**Δ after exposure**^**a**^	**Δ 20 h post-exposure**^**a**^	**Δ after exposure**^**a**^	**Δ 20 h post-exposure**^**a**^
***FA exposure***							
Leukocytes	5.45±1.36	5.72±1.34	5.17±1.17	0.27±0.53	-0.28±1.02	--	--
Neutrophils	3.02±1.15	2.99±1.03	2.64±0.75	-0.04±0.31	-0.38±0.99	--	--
Eosinophils	0.19±0.09	0.20±0.11	0.21±0.11	0.01±0.06	0.02±0.06	--	--
Lymphocytes	1.69±0.49	2.02±0.64	1.83±0.53	0.33±0.32	0.14±0.30	--	--
Monocytes	0.49±0.13	0.48±0.15	0.44±0.12	-0.01±0.13	-0.05±0.11	--	--
***DE exposure***							
Leukocytes	5.29±1.00	5.60±0.98	5.45±1.37	0.31±0.55	0.16±1.01	0.725	0.007
Neutrophils	3.00±0.81	2.91±0.63	2.89±1.04	-0.09±44	-0.11±0.93	0.474	0.139
Eosinophils	0.19±0.12	0.20±0.13	0.22±0.13	0.01±0.06	0.03±0.07	0.847	0.431
Lymphocytes	1.59±0.48	1.94±0.59	1.82±0.64	0.35±0.24	0.23±0.35	0.651	0.264
Monocytes	0.47±0.11	0.51±0.10	0.48±0.13	0.04±0.11	0.01±0.10	0.042	0.017

Values are mean ± standard deviation (SD).

*DE*, diesel exhaust; *FA*, filtered air.

^a^Δ after exposure = after exposure – before exposure; Δ 20 h post-exposure = 20 h post-exposure – before exposure.

Data on other inflammatory markers in serum and nasal lavage are shown in Table 7. We only found a significant difference between CC16 changes in DE and FA exposure. A slight increase in CC16 was observed immediately after DE exposure and returned to baseline at 20 h post-exposure for DE exposure, while it increased at 20 h post-exposure of FA exposure (p=0.016). A trend towards a greater increment of serum IL-6 for DE exposure than FA exposure was suggested at 20 h post-exposure (p=0.066). However, CRP tended to decrease more rapidly after exposure to DE than after FA (p=0.034).

**Table 7:** Effects of FA exposure and DE exposure on inflammatory markers

**Inflammatory markers**	**Concentration of markers**					**P-values for DE exposure vs. FA exposure**	
	**Before exposure**	**After exposure**	**20 h post-exposure**	**Δ after exposure**^**a**^	**Δ 20 h post-exposure**^**a**^	**Δ after exposure**^**a**^	**Δ 20 h post-exposure**^**a**^
***FA exposure***							
Serum IL-6 (pg/ml)	1.73±0.98	1.69±1.13	1.60±1.33	-0.04±0.56	-0.13±0.90	--	--
Serum IL-8 (pg/ml)	5.00±3.28	4.91±3.26	5.46±4.35	-0.09±1.33	0.46±2.48	--	--
NL IL-6 (pg/ml)	0.97±1.01	0.94±0.86	0.85±0.68	-0.03±0.93	-0.11±0.81	--	--
NL IL-8 (pg/ml)	67.0±73.3	48.5±50.5	49.9±66.6	-18.6±71.5	-17.1±49.4	--	--
NL TNF-α(pg/ml)	0.95±0.77	0.84±0.14	1.03±0.88	-0.10±0.79	0.08±1.19	--	--
NL ICAM-1(pg/ml)	76.8±87.1	76.6±101	62.0±73.9	-0.15±114	-14.7±64.0	--	--
CRP (mg/l)	1.6±1.6	1.5±1.7	1.3±1.2	-0.03±0.23	-0.27±0.54	--	--
Fibrinogen (g/l)	2.9±0.5	2.9±0.5	3.1±0.6	0.12±0.72	0.24±0.66	--	--
CC16 (μg/l)	5.93±1.98	5.84±2.20	6.73±2.38	-0.08±0.99	0.61±1.56	--	--
SP-D (μg/l)	66.9±34.0	66.7±35.0	70.8±36.1	-0.21±9.42	3.90±9.81	--	--
***DE exposure***							
Serum IL-6 (pg/ml)	1.80±1.35	1.64±1.14	1.92±1.56	-0.16±0.64	0.12±0.78	0.413	0.066
Serum IL-8 (pg/ml)	5.10±3.62	4.85±3.79	5.59±3.74	-0.25±1.90	0.49±1.82	0.666	0.940
NL IL-6 (pg/ml)	1.32±2.03	1.29±2.08	2.01±5.04	-0.03±2.35	0.70±4.64	0.983	0.247
NL IL-8 (pg/ml)	71.3±71.7	63.9±97.8	48.6±46.7	-7.40±98.4	-22.7±48.1	0.483	0.597
NL TNF-α (pg/ml)	0.82±0.00	0.90±0.37	0.98±0.79	0.08±0.37	0.16±0.79	0.312	0.715
NL ICAM-1(pg/ml)	109.7±138	93.5±142	85.3±103	-16.2±114	-24.4±74.5	0.409	0.398
CRP (mg/L)	2.0±2.2	1.9±2.0	1.6±1.6	-0.12±0.24	-0.39±0.71	0.034	0.283
Fibrinogen (g/l)	3.2±0.7	3.3±0.8	3.4±01.0	0.13±0.55	0.26±0.84	0.968	0.940
CC16 (μg/l)	6.21±2.23	6.64±3.44	6.12±2.32	0.43±2.50	-0.09±1.44	0.224	0.016
SP-D (μg/l)	63.4±34.0	61.9±32.7	66.7±32.8	-1.45±4.78	3.34±12.1	0.626	0.838

Values are mean ± standard deviation (SD).

*DE*, diesel exhaust; *FA*, filtered air; *NL*, nasal lavage.

^a^Δ after exposure = after exposure – before exposure; Δ 20 h post-exposure = 20 h post-exposure – before exposure.

## Discussion

This study was extracted from DINO project, the overall aim of which was to evaluate the combined effect of DE and traffic noise on human physiological response (including also cardiovascular responses such as heart rate variability). Although effects of traffic noise on airways have not been documented, we still initially included traffic noise in the analysis, to see if it modified the reported or observed airway effects of DE. However, no such modifying effect was found in any markers of respiratory response. Moreover, the generation of DE was highly reproducible between the sessions. Thus, for optimal statistical power, we merged the four exposures into two: exposure or no exposure to DE.

The concentration of DE in this study was nearly 300 μg/m^3^, and such levels may occur at rush hours in large city centers. We found divergent trends in PEF changes during DE and FA exposure, and a significant DE-associated PEF decrease at 75 min in healthy subjects. Self-reported symptoms and the physical examination indicated irritation of the upper airways. The increase in peripheral leukocyte counts suggests a mild systemic inflammatory response, supported also by a tendency towards an increase in serum IL-6.

To the best of our knowledge, this is the first study that shows an effect on PEF in healthy subjects. DE induced a slight but consistent reduction in PEF that was significantly different from FA exposure at 75 min. Thereafter differences were still suggested at 135 min and after exposure, approaching significance. Our interpretation is that DE caused a transient lung function change with rapid recovery. PEF was seldom measured at DE exposure in healthy volunteers, but in asthmatics exposed to DE (PM_10_ 100 μg/m^3^) for 2 h, there was a non-significant trend towards a decreased PEF immediately after exposure [33]. Similar to other studies [20,34], we performed spirometry before and after exposures and failed to find significant difference in FEV_1_ changes, although we used a longer exposure time (3 h). This also reflected that the time points of monitoring may be important. In this study, there was a 50 min delay between end of exposure and spirometry, which may allow the lung function to recover. Moreover, the subjects of this study were older (mean age about 50 years) than in other studies.

The small but significant PEF changes in healthy subjects may imply that occupational DE limit values should be reconsidered. DE is quite common in occupational settings, and some studies have indicated adverse effects on lung function at long-term occupational exposure to DE in combination with dust [10,35]. The current American Conference of Governmental Industrial Hygienists (ACGIH) Threshold Limit Values (TLVs) for respirable particulates and NO_2_ are 3 mg/m^3^ and 3 ppm as 8-hour time-weighted averages (TWAs), respectively [36]. However, the present study indicates that substantially lower exposures (PM_1_ level of 300 μg /m^3^ and NO_2_ of 1.3 ppm) to DE for only three hours will cause a transient effect on lung function.

As in previous studies [23,37], self-reported throat and eye irritation [26] increased during DE exposure, in agreement with objective signs at the physical examination. The emission of aldehydes is the most likely cause in the present study. A Swedish study found an increase in nasal irritation and lower respiratory tract symptoms at an average exposure of 260 μg/m^3^ of formaldehyde [38]. Our exposure level of formaldehyde, about 400 μg/m^3^, was higher than in these reports, and DE also contains other aldehydes such as acrolein that may cause such irritant symptoms.

In the present study, we used nasal lavage for measurement of the acute cytokines (IL-6, IL-8, TNF-α, ICAM-1) to define the timing of inflammatory response in the nasal cavity instead of the more invasive bronchoalveolar lavage and bronchoscopy techniques. However, such repeated sampling of airway fluid could have the drawback of washout effect [30,39], resulting in lower concentration of biomarkers in subsequent lavages and then diminishing real differences between DE and FA exposures. The only finding in nasal lavage in this study was a slight increase (not statistically significant) of IL-6 after DE exposure at 20 h post-exposure. Other cytokines (IL-8, TNF-α, ICAM-1), on the contrary, remained unchanged. Nasal lavage has not been widely investigated in chamber studies of DE exposure, Blomberg et al. [40] only found an increase in ascorbic acid concentration in nasal lavage after diesel exposure (1 h, PM 300 μg/m^3^). In other in vivo studies, intranasal challenge with allergen + DE in atopic subjects was found to increase IgE, IL-4 and histamine concentrations in nasal lavage [41,42], but concentrations of IL-6, IL-8 were not altered by intranasal challenge with DE alone in healthy volunteers [43].

CC16 is an anti-inflammatory protein secreted by the Clara cells located in the bronchioli [44], and the transfer to serum is increased when the permeability of the lung epithelial barrier is compromised [45]. Two chamber studies found increased serum CC16 after exposure to wood smoke at PM mass concentrations similar to those in the present study [46,47]. CC16 levels were, however, seldom reported at DE exposure in previous chamber studies. In this study, the slight increase of serum CC16 immediately after DE exposure was not statistically significant and levels returned to baseline at 20 h post-exposure. The reason for the increase in serum CC16 the morning after FA exposure is unclear.

DE exposure, at the moderate levels used in this study, induced a systemic inflammatory response in peripheral blood. Previous experimental studies showed white cell and proinflammatory cytokines changes in bronchoalveolar lavage and bronchial biopsies, but rarely in peripheral blood. Total leukocyte counts were higher after 3 h exposure to both DE and FA in the afternoon than before exposure in the morning, which reflects the normal circadian variation [48]. However, the total leukocyte counts remained increased at 20 h post-exposure after DE exposure, but not after FA exposure, which suggests a slight systemic cellular inflammatory response after exposed to DE. Changes in pro-inflammatory cytokines in peripheral blood were also identified. The trend towards an increase of serum IL-6 (p=0.07) after DE exposure at 20 h post-exposure, which was also observed by Tornqvist et al. [49], may represent a pro-inflammatory systemic response. However, consistent with other controlled DE chamber studies performed in either healthy volunteers [50,51] or individuals with chronic obstructive pulmonary disease [52], we did not find a DE-associated increase in CRP. The follow-up time in the present study perhaps has been too short to detect a change in CRP.

## Conclusion

In summary, this study shows that short-term exposure to DE at 300 μg/m^3^ can cause irritation in upper airways, along with a temporary decline in PEF in healthy subjects, after only 75 min into exposure. Additionally, the increase in leukocyte cell counts in peripheral blood indicates that these levels of DE exposure also cause a systemic inflammatory response.

## Abbreviations

DE: Diesel exhaust; PM: Particulate matter; PEF: Peak expiratory flow; FA: Filtered air; VAS: Visual analogue scale; MWPFM: Mini-Wright Peak Flow Meter; FVC: Forced vital capacity; FEV1: Forced expiratory volume in one second; MCA1: Minimal cross-sectional area between 0–22 mm; MCA2: Minimal cross-sectional area between 22–45 mm; CRP: C reactive protein; IL: Interleukin; ICAM: Intercellular adhesion molecule; TNF: Tumor necrosis factor; CC16: Clara cell protein; SP-D: Surfactant protein D; OR: Odds ratio; ACGIH: American Conference of Governmental Industrial Hygienists; TLVs: Threshold Limit Values; TWAs: 8-hour time-weighted averages

## Competing interests

The authors declare that they have no competing financial interests

## Authors’ contributions

AW, AG and MA designed the project and performed the chamber exposure. AG and AW generated the aerosol. AW provided exposure characteristics. JN and MA were responsible for the medical examinations. BJ and MK measured biomarkers. YX, LB, AA, MK and MA analyzed the data and interpreted the results. The manuscript was written by YX and revised critically by LB and MA. All authors read, corrected and approved the manuscript.
